# An Evaluation of Leaf Biomass : Length Ratio as a Tool for Nondestructive Assessment in Eelgrass (*Zostera marina* L.) 

**DOI:** 10.1100/2012/543730

**Published:** 2012-05-01

**Authors:** Hector Echavarria-Heras, Elena Solana-Arellano, Kun-Seop Lee, Shinya Hosokawa, Ernesto Franco-Vizcaíno

**Affiliations:** ^1^Centro de Investigación Científica y de Educación Superior de Ensenada, Km 107 Carretera Tijuana, 22860 Ensenada, BCS, Mexico; ^2^Department of Biology, Pusan National University, Pusan, Republic of Korea; ^3^Coastal and Estuarine Environmental Research Group, Port and Airport Research Institute, Nagase, Yokosuka, Kanagawa, Japan; ^4^Department of Science and Environmental Policy, California State University Monterey Bay, 100 Campus Center, Seaside, CA 93955, USA

## Abstract

The characterization of biomass and its dynamics provides valuable information for the assessment of natural and transplanted eelgrass populations. The need for simple, nondestructive assessments has led to the use of the leaf biomass-to-length ratio for converting leaf-length measurements, which can be easily obtained, to leaf growth rates through the plastochrone method. Using data on leaf biomass and length collected in three natural eelgrass populations and a mesocosm, we evaluated the suitability of a leaf weight-to-length ratio for nondestructive assessments. For the data sets considered, the isometric scaling that sustains the weight-to-length proxy always produced inconsistent fittings, and for leaf-lengths greater than a threshold value, the conversion of leaf length to biomass generated biased estimations. In contrast, an allometric scaling of leaf biomass and length was highly consistent in all the cases considered. And these nondestructive assessments generated reliable levels of reproducibility in leaf biomass for all the ranges of variability in leaf lengths. We argue that the use of allometric scaling for the representation of leaf biomass in terms of length provides a more reliable approach for estimating eelgrass biomass.

## 1. Introduction

Eelgrass (*Zostera marina*) is a widespread seagrass species that plays an important role in shallow and nearshore ecosystems. This temperate macrophyte is distributed in Northern Hemisphere habitats from the Arctic Circle to the Tropic of Cancer [1], where it plays an important role as a nursery for fish and as a substrate for attached algae and epifauna [2, 3]. By fixing large amounts of carbon through photosynthesis, this cosmopolitan seagrass species also plays a fundamental trophic role, sustaining detrital food chains and other secondary producers [4]. Eelgrass also helps in the remediation of contaminated sediments [5] by filtering and retaining nutrients from the water column [6] and contributing to the stabilization of sediments [7]. Moreover, eelgrass meadows reduce erosional forces by stumping wave energy, thus promoting the stabilization of adjacent shorelines [8, 9].

The variability in eelgrass biomass constitutes a dynamic link between its structural and trophic roles, because changes in the amount of organic carbon that can be fixed modulate the structure of the habitat for the associated biota. These organisms are affected in different ways when changes in biomass occur seasonally or unpredictably [10]. Therefore, accurate measurements of the standing crop and productivity of eelgrass constitute an important input for evaluating the ecological functions and values of this important seagrass species [11].

Growth in seagrasses occurs through the expansion of modules formed by rhizome segments, which have bundles of attached leaves and roots. Because every leaf produced corresponds to the production of a rhizome node, it is reasonable to assume that eelgrass growth and leaf formation are equivalent processes [12]. This conspicuous feature has encouraged efforts to estimate the growth of eelgrass, as well as that of other seagrasses with ribbon-like leaves, by measuring leaf growth. These estimations are customarily made by using the leaf-marking technique [13], and Sand-Jensen [14] modified Zieman [13] original method and proposed a technique for the assessment of leaf growth in *Zostera marina* in which leaves are marked with permanent ink at a fixed distance above the sheath of an older leaf.

Other authors modified Zieman's original method by using a mark produced by a puncture at a reference point in the shoot [15–20]. However, these variations in Zieman's approach assume that growth amounts to the weight of tissue produced between a fixed reference point normally placed at the top of the sheath and the position of the mark after leaf elongation [19]. The original leaf-marking technique has been considered to provide reliable estimates of productivity, thus explaining its use in many seagrass species [21]. But tissue damage caused by marking may also influence subsequent estimations of growth [19]. And this methodology cannot account for the leaf material produced within the sheath during the elongation interval [22, 23]. It has been also pointed out that leaf marking can underestimate growth because the maturation process of seagrass leaves involves cell expansion and an increase in leaf mass (leaf weight per unit area) that is not measured by the weight of newly produced leaf tissue [11, 24, 25].

 Short [25] developed the elongation mass method, which modifies the conventional leaf-marking procedure by changing the reference point placed above the sheath and puncturing the shoot at a predetermined distance above the meristem within the sheath. In order to correct the underestimation attributable to leaf growth assessments based on the weight of immature leaf sections from newly grown leaf tissues, the elongation mass method accounts for leaf elongation and weight gain that are part of total leaf growth. Growth in biomass is calculated by multiplying the leaf elongation rate by the leaf weight-to-length ratio (mg cm^−1^) of mature leaf material [11]. The notions of a weight-to-length ratio of mature leaf material, along with that of the plastochrone interval (the time period between the development of two successive leaves), are at the core of the plastochrone method for eelgrass growth assessments [11]. This procedure provides a much simpler alternative to previous time-consuming techniques that were based on traditional leaf marking. Growth is captured by using the weight of a mature leaf as a surrogate for all growing tissue in a shoot over a given plastochrone interval. In effect, measuring the length of mature leaves and converting to leaf weight by using a weight-to-length ratio can be considered as a nondestructive method for determining production of leaf biomass [26].

 Our previous results showed that an allometric representation of eelgrass leaf biomass in terms of length is highly consistent [27], whereas the leaf weight-to-length ratio proxy is sustained by an isometric scaling of leaf biomass in terms of length; therefore the suitability of either approach has to be substantiated on the basis of model selection criteria, which has not been yet produced. In the present research we have made an attempt to fill this gap. Using available data we produced a statistical evaluation of the reliability of nondestructive leaf biomass assessments obtained by means of both a weight-to-length ratio and through an allometric scaling of leaf biomass in terms of length as proxies for direct leaf biomass estimations.

## 2. Data

We analyzed an extensive data set composed of 6319 individual eelgrass leaf biomasses and their associated lengths which were collected from different populations in Punta Banda (31°43′–46′ N, 116°37′–40′ W) and San Quintin Bay (30°24′–30°37′ N, 115°56′–116°01′ W) estuaries in Baja California (Mexico), Jindong Bay (35°06′ N, 128°32′ E) in South Korea, plus similar data produced in a mesocosm experiment (35°13.7′ N, 139°43.2′ E) in Japan.

## 3. Formal Methods

 In what follows we will let *w* denote the biomass (g) and *l* the length (mm) of a *Zostera marina* leaf. For discretely obtained leaf biomass data, we used the least squares method and fitted the allometric model

(1) $$\begin{array}{c} w=al^{b}, \end{array}$$

where *a* and *b* are positive constants known, respectively, as the normalization constant and the allometric exponent. For purposes of comparison we also consider an isometric scaling of leaf biomass and length. This is formally expressed as

(2) $$\begin{array}{c} w=cl, \end{array}$$

with *c* a positive constant.

The function

(3) $$\begin{array}{cc} \theta \left(l\right) & =\{\begin{array}{cc} l\left(c-al^{b-1}\right) & \text{for}\;b\neq 1,\\ 0 & \text{for}\;b=1 \end{array} \end{array}$$

gives the deviation of leaf weight values calculated by means of the isometric model of (2) relative to those produced by the allometric model of (1). Moreover, for *b* ≠ 1 the line through the origin, which is linked to the isometric model, intersects the curve depicted by the allometric model at the origin and at a nonvanishing threshold value *l*_∗_ given by

(4) $$l_{*}={\left(\frac{c}{a}\right)}^{1/\left(b-1\right)}.$$

For *b* > 1 and *l* bounded above by *l*_∗_, leaf biomass values *w* that are calculated by means of (2) will lie above those assigned by (1) and consequently we will have positive values for *θ*(*l*). Conversely, for *l*_∗_ values beyond *l*_∗_, leaf biomass values assigned by the nonlinear model of (1) will lie above those assigned by the isometric model of (2) and will produce negative values for *θ*(*l*). For *b* < 1 the behavior of *θ*(*l*) reverses. Moreover for *b* ≠ 1 the derivative of *θ*(*l*) becomes

(5) $$\;\begin{array}{c} \frac{d\theta }{dl}=c\left(1-bl_{*}^{-(b-1)}l_{}^{\left(b-1\right)}\right). \end{array}$$

Hence, the maximum absolute deviation *θ*_max⁡_ between the isometrically and allometrically calculated values of *w* is attained at a leaf length value *l*_*θm*_ which is given by

(6) $$\begin{array}{c} l_{\theta m}=l_{*}b^{-(1/\left(b-1\right))}. \end{array}$$

Moreover for *b* ≠ 1 we have 0 < *l*_*θm*_ < *l*_∗_ and

(7) $$\begin{array}{c} \theta_{\max}={\left(\frac{c}{ab}\right)}^{b/\left(b-1\right)}a\left|b-1\right|. \end{array}$$

Hence if *θ*_max⁡_ takes on suitably small values and if an appropriately large proportion of leaf length values lie in the region 0 < *l* < *l*_∗_, we might expect great similarity between values of *w* predicted by the allometric model of (1) and the isometric model of (2). Beyond this threshold, values of *w* calculated by means of (1) will increase at a nonconstant rate, and for suitably large values of *l*, these can be expected to significantly diverge from those assigned by the model of (2).

 When individual leaf dry weights were not available because biomass data had been obtained as shoot-level aggregates, (1) and (2) could not be fitted to obtain the allometric parameters *a* and *b* or the value of the isometric normalization constant *c*. But the dry weight of each leaf in a given shoot can nonetheless be considered as a random variable and thus can be expressed in terms of the basic model of (1). Hence, for the biomass of a particular shoot which is denoted here by the symbol *w*_*s*_ we have the aggregated allometric equation

(8) $$\begin{array}{c} w_{s}=\overset{n_{s}}{\underset{k=1}{\sum }}al_{k}{\left(t\right)}^{b}, \end{array}$$

where *l*_*k*_(*t*) denotes the length of the *k*th leaf and *n*_*s*_ stands for the number of leaves in the shoot being considered. Meanwhile, for the isometric scaling case we have

(9) $$\begin{array}{c} w_{s}=\overset{n_{s}}{\underset{k=1}{\sum }}cl_{k}\left(t\right). \end{array}$$

## 4. Results

**Figure 1:**
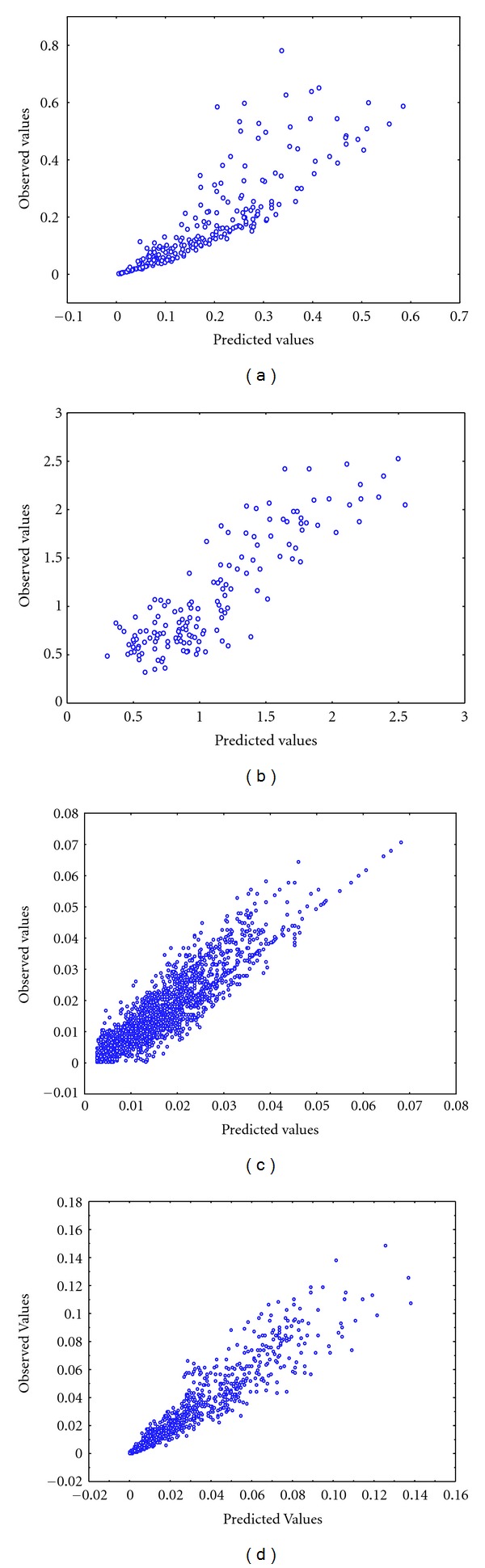
Observed versus predicted values for the fitting of the allometric model for each site. (a) Mesocosm, (b) Jindong Bay, (c) Punta Banda estuary, and (d) San Quintin Bay.

**Figure 2:**
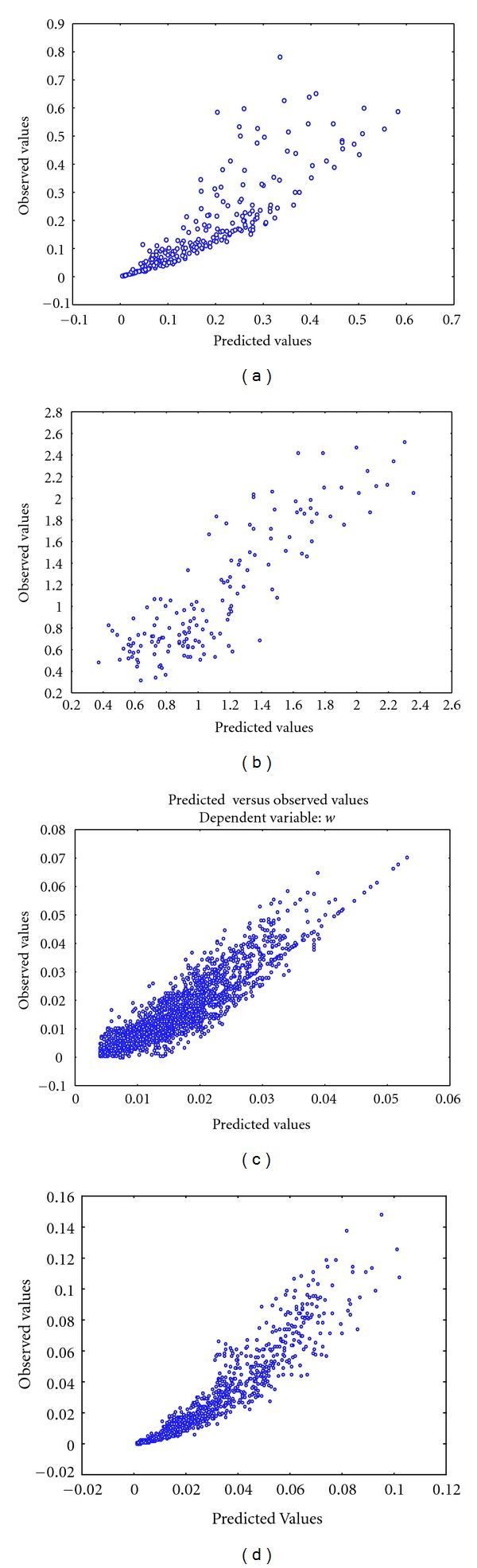
Corresponding observed versus predicted values for the fitting of the isometric model for each site. (a) Mesocosm, (b) Jindong Bay, (c) Punta Banda estuary, and (d) San Quintin Bay.

The values of the parameters *a* and *b*, their standard errors, determination coefficients (*R* ^2^), and the values of the concordance correlation coefficient (CCC) of reproducibility $\left(\hat{\rho }\right)$ [28] resulting from the fittings of the allometric models of (1) or (8) are presented in Table 1. Correspondingly in Table 2 we present the values of the parameter *c*, standard errors, determination coefficients (*R* ^2^), and $\hat{\rho }$ values which were obtained by fitting either (2) or (9) for an isometric scaling of leaf biomass and length for each site. The predicted versus observed values for the allometric and isometric models are displayed in Figures 1 and 2, respectively.

**Figure 3:**
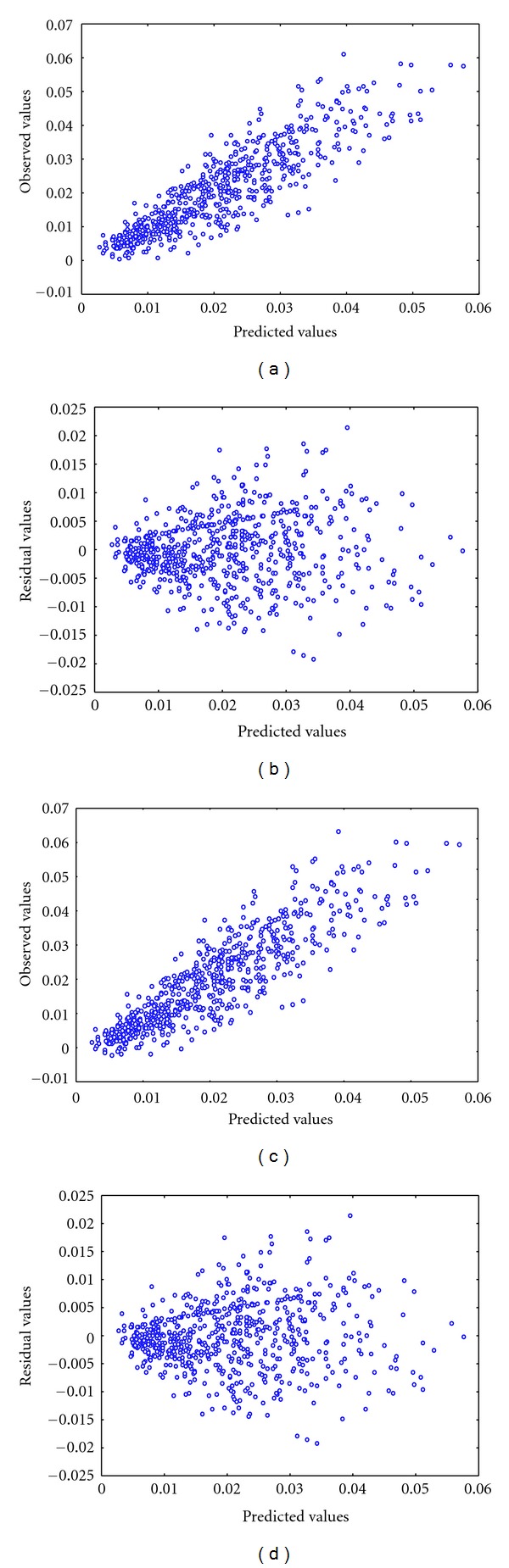
Comparison of observed versus predicted values (a) and the disposition of residuals (b) for the fit of the allometric model for whole developed leaves (leaf-3) in Punta Banda data. Corresponding comparison of observed versus predicted values (c) and the disposition of residuals (d) for the fit of the isometric model for whole developed leaves (leaf-3) in Punta Banda data.

**Figure 4:**
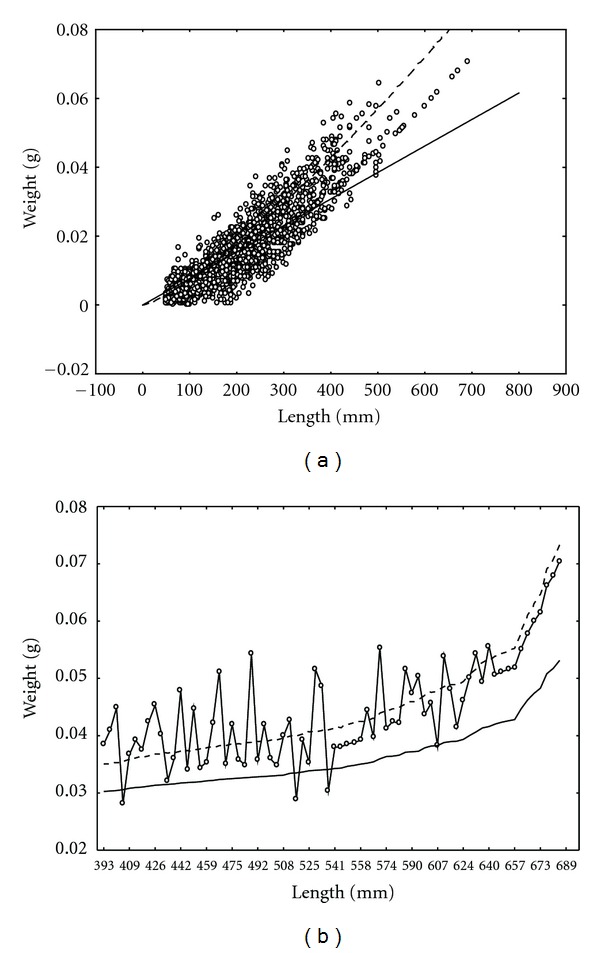
(a) Spread of raw data and plots produced by the fitted allometric (dashed lines) and isometric (continuous lines) models. (b) Subset of data containing leaf length values from 390 mm to 690 mm.

For the Punta Banda data a whole developed leaf (leaf-3) was identified. This leaf has been considered as a proxy for mature leaf material [11]. We found a determination coefficient of 0.80 for the allometric equation (1). For the fitted parameters we found *a* = 0.000016 ± 0.000003 and *b* = 1.29 ± 0.03, with $\hat{\rho }=0.88$. For the isometric model we found a determination coefficient of 0.93, *c* = 0.00009 ± 0.000001, and $\hat{\rho }=0.80$. Figures 3(a)–3(d) show the comparison of observed versus predicted values and the disposition of residuals of these fits. We found no significant differences between the observed means for leaf biomass and the allometrically projected values (*P* = 0.56), but significant differences were found between means of observed and isometrically calculated values (*P* < 0.00001). Moreover we performed a lack-of-fit test for the isometric model and found a significant lack of fit with *F*_370_^554^ = 1.92 and *P* < 0.00001. Inspection of the spread of raw data in the plots produced by the fitted models (Figure 4(a)) revealed that values predicted by both models behave similarly for lengths from 0 to 120 mm, which we identified as the threshold value *l*_∗_, at which the intersection of the plots of the fitted models occurs (cf. (4)).

Beyond *l*_∗_ the plots generated by the allometric and isometric models start to deviate, and their projected values become markedly different. To elucidate this thoroughly, we selected a subset of data containing leaf length values from 390 mm to 690 mm (see Figure 4(b)). The values projected by fitting the allometric model cross through the middle of the dispersion of observed values, while almost all values of leaf dry weight projected by the isometric model lie below the observed values. This shows that beyond the leaf-length threshold value *l*_∗_, bias can be expected in leaf biomass values calculated by means of a leaf weight-to-length ratio.

Results of modeling based on data from mature leaf materials obtained at Punta Banda were validated by using the four additional leaf data sets. Using the values of parameters *a*, *b*, and *c*, which were fitted at each site, we calculated the respective values of the leaf length threshold *l*_∗_. And for each site we obtained the percentages of leaf length values that placed before and after the leaf length threshold, that is, *l* < *l*_∗_ and *l* > *l*_∗_ (Table 3). Similarly for all sites, we used (7), and the fitted values of the parameters *a*, *b*, and *c*, to calculate the respective maximum absolute deviation values *θ*_max⁡_ (Table 3). That *θ*_max⁡_ is always smaller than any of the standard errors of fits (Tables 1 and 2) implies that in the region 0 < *l* < *l*_∗_ leaf weight values calculated by using either model will be indistinguishable. But while the allometric model presented good consistency in residuals, the isometric model failed all the assumptions for a good fit, and the isometric model attains its minimum CCC value for all sites.

Data for Jindong Bay, Punta Banda, and San Quintin Bay showed that 82%, 70%, and 80% of leaf length values were less than the respective *l*_∗_ values. But even though it can be expected that most data can be well represented by both models, we nevertheless found a significant lack of fit for the isometric model at these sites (*P* < 0.05), while the allometric model was highly consistent. The mesocosm presented large variability in leaf weights and only 20% of leaves had lengths smaller than *l*_∗_; this produced a significant lack of fit for the isometric model (*P* < 0.05). On the other hand, residuals for the fit of the allometric model showed a slight departure from homoscedasticity (*P* = 0.049), while fulfilling all the other residual characteristics for a good fit and thus sustaining the consistency of this model.

For all four data sets, the selection of the allometric model over the isometric alternative is supported by the values of CCC and the behavior of residuals (Tables 1 and 2). This conclusion was reinforced by values of the Akaike Information Criterion (AIC), which were smaller for the allometric than the isometric model for all sites (Table 4). And since the difference in units between both AIC indexes is >4 (in fact >10 in most cases) we conclude that the isometric model fails to explain the structural variation in the data in a consistent way [29] and thus cannot reliably represent the relationship between leaf weight and length in eelgrass.

## 5. Discussion

Calculating eelgrass leaf biomass in terms of a leaf weight-to-length ratio has required the identification of an isometric scaling of leaf biomass in terms of length. But previous results [27], as well as those reported here, show that an allometric representation of eelgrass leaf biomass in terms of length is highly consistent. Therefore the presumed adequacy of an isometric model for the representation of eelgrass leaf biomass in terms of length must be properly substantiated. Indeed, in accordance with the discussion on (7), both the allometric and isometric models can be fitted to a particular data set. Ambiguity between the models could arise mainly if the calculated value of *θ*_max⁡_ takes a value comparable to the minimum of the standard errors associated with these fits, and if the value of *l*_∗_ is suitably large. This would result in similar predictions by both models within the region 0 < *l* < *l*_∗_. Nonetheless, the values of leaf biomass predicted by these models can be expected to diverge for lengths beyond the threshold *l*_∗_. Moreover, as shown by (2), the rate of change of leaf biomass in terms of length remains constant for the isometric model, while (1) indicates that it changes with length for the allometric alternative. Hence it is the contribution of leaf lengths beyond the threshold *l*_∗_ which mainly explains the consistency of the allometric alternative. But even though the allometric model explains the structural variation of the data in a consistent way, for some data sets, model ambiguity caused by a small *θ*_max⁡_ value, and a suitable proportion of observed leaf lengths lying in the region 0 < *l* < *l*_∗_ could still produce a high determination coefficient value for the fit of the isometric model. But this value by itself, without an evaluation of the behavior of residuals, would fail to adequately fulfill the criteria for selection of the isometric model. And even if all, or most, of the leaf length values do lie in the interval 0 < *l* < *l*_∗_, *θ*_max⁡_ might still be large enough as to make the intrinsic nonlinearity of a true allometric dependency of leaf dry weight on length to induce a lack of support for the isometric assumption. This lack of support was corroborated by the results presented here, which show that the isometric scaling produced inconsistent fittings for all four available data sets. And we show here that, regardless of a high determination coefficient value for the fitting of the isometric model, the use of the related leaf weight-to-length ratio as an indirect device for inferring leaf biomass may still produce biased results, particularly for leaf lengths beyond the threshold *l*_∗_. And since the allometric scaling of leaf biomass and length was found to be highly consistent for the whole range of variation in leaf lengths, the resulting values of leaf weights calculated allometrically produced higher CCC values for the reproducibility of measured leaf biomasses. We therefore argue that the allometric model better fulfills the criteria for model selection and that for leaf length values beyond the threshold *l*_∗_, the conversion of leaf length to biomass based on a leaf weight-to-length proxy can be expected to underestimate observed leaf weights. The larger the percentage of leaf length values greater than *l*_∗_, the larger the bias that can be expected. For example, for the mesocosm data set, the isometric model could be underestimating about 80% of observed values and miscalculating the observed weight values by 30%, 20%, and 18%, for San Quintin, Punta Banda, and Jindong Bay, respectively. We have pointed out the importance of local and regional factors in the determination of the values of the involved allometric parameters [30]; this is also expected to hold for the isometric model sustaining a weight-to-length ratio for a particular population. Therefore, in general the percentages of miscalculation due to the use of this proxy will depend on the values of the parameters fitted for the addressed population.

The loss of eelgrass habitat has been noted worldwide, with catastrophic losses within the past few decades [31–33]. These concerns have driven efforts to conserve eelgrass populations, and several workers have relied on transplantation projects as a way to restore lost habitats [34–37]. Evaluation of the success of restoration efforts requires the use of nondestructive approaches like the plastochrone method. But our results indicate that, when addressing such assessments methodology, for unbiased estimations, using of an allometric proxy for the conversion of leaf length to biomass, instead of the traditional leaf biomass-to-length ratio, should be highly recommended.

## Figures and Tables

**Table 1:** Values of the parameters *a* and *b*, standard errors, *R* ^2^, $\hat{\rho }$ and standard error of the fit resulting from the fittings of the allometric models of (1) or (8).

Study site	*a*	*b*	*R* ^2^	$\hat{\rho }$	Std error of fit
Mesocosm	0.000104 ± 0.041	1.1628 ± 0.057	0.74	0.80	0.082
Jindong Bay	0.000172 ± 0.000058	1.206 ± 0.054	0.77	0.88	0.270
Punta Banda	0.000015 ± 4*E*−12	1.26 ± 0.0275	0.85	0.91	0.004
San Quintin	0.00001 ± 0	1.410012	0.92	0.91	0.006

**Table 2:** Values of the parameter *c*, standard errors, *R* ^2^, $\hat{\rho }$ and standard error of the fit resulting from the fittings of the isometric models of (2) or (9).

Study site	*c*	*R* ^2^	$\hat{\rho }$	Std error of fit
Mesocosm	0.00032 ± 0.000008	0.86	0.83	0.083
Jindong Bay	0.00062 ± 0.0001	0.74	0.84	0.286
Punta Banda	0.000077 ± 4 × 10^−7^	0.91	0.86	0.005
San Quintin	0.0001 ± 0.000001	0.91	0.86	0.008

**Table 3:** Values of the leaf length threshold *l*_∗_ using the values of parameters *a*, *b*, and *c*, fitted at each site. Percentages of *l* < *l*_∗_ and *l* > *l*_∗_ and values of *θ*_*max*_ for all sites.

Site	*l*_∗_	*l* < *l*_∗_	*l* > *l*_∗_	*θ*_max⁡_
Mesocosm	175.3	20	80	0.018
Jindong Bay	474.2	82	18	0.019
Punta Banda	119	70	30	0.0035
San Quintin	274.8	80	20	0.0035

**Table 4:** Comparison of the Akaike Information Criterion (AIC) indexes between the isometric and the allometric model for all sites.

Site	AIC_isometric_	AIC_allometric_	Difference in units
Mesocosm	−1207	−1212	6
Jindong Bay	−374	−387	13
Punta Banda	−38348	−39962	1614
San Quintin	−20471	−21690	1218
